# Hybrid flagellin as a T cell independent vaccine scaffold

**DOI:** 10.1186/s12896-015-0194-0

**Published:** 2015-08-12

**Authors:** Kaila M. Bennett, Ronald D. Gorham, Veronica Gusti, Lien Trinh, Dimitrios Morikis, David D. Lo

**Affiliations:** Division of Biomedical Sciences, School of Medicine, University of California Riverside, California, 92521 USA; Bioengineering Interdepartmental Graduate Program, California, USA; Department of Bioengineering, University of California Riverside, California, 92521 USA

## Abstract

**Background:**

To extend the potency of vaccines against infectious diseases, vaccines should be able to exploit multiple arms of the immune system. One component of the immune system that is under-used in vaccine design is the subset of B cells known to be capable of responding to repetitive antigenic epitopes and differentiate into plasma cells even in the absence of T cell help (T-independent, TI).

**Results:**

To target vaccine responses from T-independent B cells, we reengineered a bacterial Flagellin (FliC) by replacing its exposed D3 domain with a viral envelope protein from Dengue virus (DENV2). The resulting hybrid FliC protein (hFliC) was able to form stable filaments decorated with conformationally intact DENV2 envelope domains. These filaments were not only capable of inducing a T cell-dependent (TD) humoral antibody response, but also significant IgM and IgG3 antibody response in a helper T cell repertoire-restricted transgenic mouse model.

**Conclusions:**

Our results provide proof-of-principle demonstration that a reengineered hybrid FliC could be used as a platform for polymeric subunit vaccines, enhancing T cell-dependent and possibly inducing T-independent antibody responses from B-1 B cells as well.

**Electronic supplementary material:**

The online version of this article (doi:10.1186/s12896-015-0194-0) contains supplementary material, which is available to authorized users.

## Background

Adaptive immune antibody responses rely primarily on the interaction between T helper cell populations promoting B cell activation, isotype switching, and development of plasma cells producing immunoglobulin, with subsequent pathogen opsonization/viral neutralization [1–3]. However, in T cell-deficiency settings such as AIDS, this TD mode of antibody production is ineffective at conferring protection to the host. Fortunately, “innate-like” B cells, which include splenic marginal zone B cells (15 % of total B cells) and peritoneal B1 B cells (B1a and B1b, 3 % of total of B cells), can become activated after recognition of repetitive or polymeric antigenic epitopes causing B cell receptor (BCR) clustering, independently of T helper (Th) subset (TI: [4, 5]). Efficient BCR clustering is crucial for the generation of a TI antibody response and in fact it has been determined that 10–20 BCRs must be crosslinked in order to mount a TI response [6, 7]. On resting B cells, BCRs are spaced approximately 35 nm apart, requiring TI antigens to be at least 500 nm in length [7]. Despite the chemical diversity of TI antigens, most TI antigens repetitive epitopes are spaced 5–10 nm [6, 8]. Once a TI B cell has become activated, its antibody response is largely characterized by the production of IgM and certain IgG isotypes (IgG3 and possibly IgG1: [9]). B1 B cells have also been found to undergo class switch recombination from IgM to IgA. Although the anatomic site of this isotype switch remains elusive, this suggests that a significant proportion of IgA may be generated in a TI manner [10].

While many bacterial capsular polymeric components have been studied for their ability to stimulate a TI antibody response, it remained to be tested whether bacterial flagellin (FliC), a major antigen present on many commensal and pathogenic microbes, can be exploited as a scaffold for TI vaccines [11, 12]. Indeed, one study suggested that polymeric flagellin, like its monomeric form, relies on T cell help to stimulate a humoral immune response. However, this study did not include IgM and IgG3 (two major antibody isotypes indicative of TI response) in their analysis [13]. FliC from *Salmonella serovar enterica typhimurium (S. Typhimurium)* is the major protein making up the flagellum (11-fold symmetry) and can grow to 15 μm in length [14]. *In vitro* studies revealed that FliC is able to form filaments hundreds of nanometers in length [15]. This is a critical feature for the success of TI vaccines, as it has been suggested that a TI antigen needs to be at least 500 nm in length to produce a significant TI response.

**Fig. 1:**
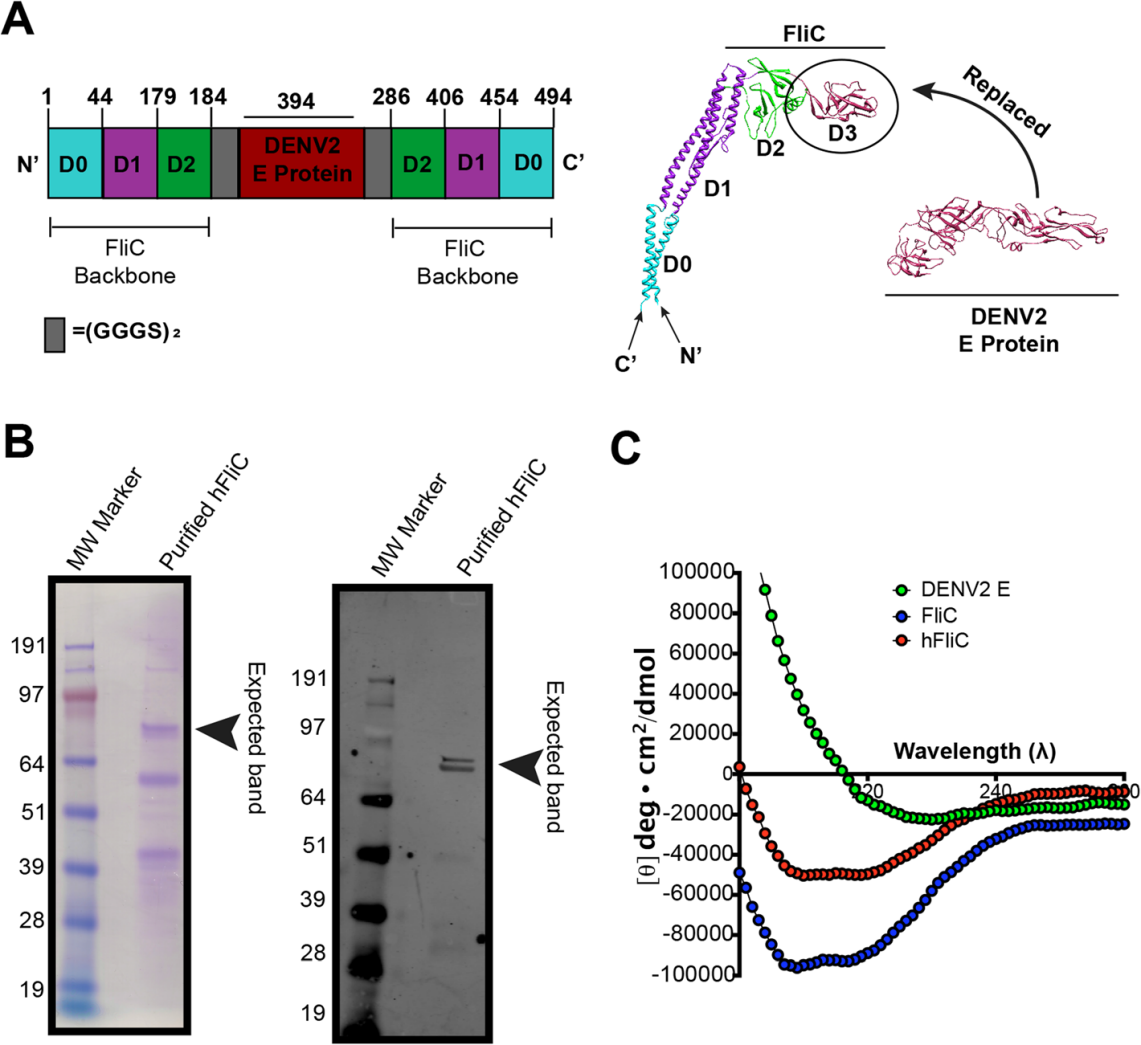
Design and analysis of hybrid flagellin protein. **a** Schematic of the construction and design of hybrid flagellin (hFliC). The D3 domain from the native FliC protein was deleted (residues 185 to 285). DENV2 E plus GS linkers flanking the termini were inserted to replace the D3 domain of FliC. The final gene product was cloned into the pENTR plasmid for baculovirus expression of the hybrid protein. **b** Protein expressed by baculovirus expression system was checked for purity and anticipated molecular weight (~85 kDa) using both Coomassie stain (left panel, which labels all proteins) and Western blot (right panel). In the Western blot, protein was probed with an antibody against the His-tag under denaturing conditions. Major bands from both blots indicate appropriate band size, however some degradation products were present in Coomassie gel; by densitometry the predicted hFliC band comprised approximately 15 % of total protein. The double band in the Western blot suggests a proteolytic cleavage very near the N-terminus. **c** Circular dichroism (CD) was used to assess secondary structural feature of the hFliC protein. CD spectra of FliC (blue curve) exhibit a characteristic α-helical region, while DENV2 spectra (green curve) exhibit a large β-sheet and coil regions. hFliC CD spectra (red curve) was essentially a hybrid of the two proteins

FliC is a four-domain (D0, D1, D2, D3) protein that structurally resembles the Greek letter Γ (Fig. 1), and has been well characterized for its adjuvant activity due to its Toll-like receptor 5 (TLR5) and Nod-like receptor CARD domain-containing protein 4 (NLRC4) binding sites located in its D1 domain, a domain also responsible for polymerization [14, 16–18]. In polymerized FliC filaments, the D3 domain of FliC is completely solvent exposed, while other domains remain mostly buried within the inner core. Moreover, based on the crystallographic structure of the Filament the D3 domains are spaced ~5 nm apart. Muskotal *et al.* [19] found that as a monomer the D3 domain is the most stable portion of FliC, where D0 and D1 completely lose their α-helical secondary structure, corroborating previous structural studies. Moreover, by eliminating D3, this study found that D3 was dispensable for the overall stability of the flagellum filament, marking D3 as a possible insertion site for novel antigenic determinants.

The polymeric nature of FliC filaments intrinsically provides the key feature needed for stimulating a potent TI response, so we wanted to test its ability as a vaccine scaffold for TI antigens. Our approach was to test the FliC D3 site for insertion of a fully independent conformational domain, and polymerizing the engineered protein as a vaccine target. We engineered native FliC by replacing its entire D3 domain with the full length sequence of the Envelope glycoprotein (E) from Dengue virus serotype 2 (E), bridged by flexible linker sequences. This allowed for the antigen portion of the hybrid FliC (hFliC) protein to be fully exposed in polymerized filaments, enabling engagement of the antigen with the B cell antigen receptors for BCR crosslinking and subsequent TI stimulation. Our studies showed that the hFliC filaments were able to stimulate a potent antibody response; more importantly, the filament induced a significant response in a helper T cell-restricted mouse model. Thus, the study presented herein describes the use of FliC as a novel scaffold for the design of potential TD/TI vaccines.

## Results

### Design and characterization of hFliC protein

To take advantage of the solvent exposed location of the D3 domain of FliC, we replaced this domain with a vaccine antigen domain, so that the surface of the polymerized hybrid protein filament would present polymeric vaccine antigen instead of flagellin epitopes. Thus, the flagellin D3 domain from residues 185 to 285 were removed and replaced with the extracellular domain of the DENV2 envelope protein (Dengue 2 E: Fig. 1a). DENV2 E is one of three structural proteins produced by the Dengue 2 virus (there are 4 identified Dengue serotypes, sharing ~70 % genome homology) and forms an icosahedral scaffold at the virion surface. DENV2 E mediates the fusion of virus to host cells and as such has been extensively studied for its antigenicity as a DENV2 vaccine [20]. In order to maintain the conformational independence of this domain, we added Gly-Ser flexible linkers to join FliC to the N- and C- terminus of DENV2 E. The resulting hybrid protein is therefore comprised of a central independent DENV 2 E protein domain, flanked by the two independent N-and C- terminal helical domains of FliC; folding of the E protein domain will help stabilize the helical domains of FliC, although further stabilization occurs in the polymerized filament when interactions between helical domains of the monomers interact. Finally, a C-terminal His_6_-tag was added for production and purification purposes. The hybrid FliC protein (hFliC) was produced using a baculovirus expression system, purified (Fig. 1b), and subsequently assayed for appropriate structure and function. After His-tag enrichment, the hFliC band is clearly evident although comprising only about 14 % of total protein; Western blot indicates a possible proteolytic cleavage near the N-terminus.

Circular dichroism (CD) was used to assess the secondary structure of purified hFliC protein (Fig. 1c), and compared to that of free FliC and DENV2. Quantification of percent secondary structure from the CD spectra revealed that FliC (protein size and purity confirmed: Additional file 1: Figure S1A) was largely α-helical, Meanwhile, DENV2 (protein size and purity confirmed: Additional file 1: Figure S1b) had a larger β-sheet region, consistent with previous published spectra, and was in good agreement with computationally determined secondary structure percentages based on crystallographic structures (Table 1: [21]). The hybrid hFliC exhibited a spectrum of mixed α-helical and β-sheet character (Fig. 1c). Taken together these data suggest that whole protein antigens can be inserted into FliC backbone without jeopardizing the native secondary structure of either protein.

**Table 1:** Quantification of secondary structure from CD spectra

% of Secondary Structure	α-helix	β-Sheet	Random coil
FliC			
Computationally determined^a^	43.00	21.00	35.00
Experimentally determined^b^	71.10	7.20	21.70
DENV2 E protein			
Computationally determined^a^	7.00	48.00	55.00
Experimentally determined^b^	2.03	39.21	58.00
hFliC			
Experimentally determined^b^	56.00	18.40	25.60

^a^Calculated using Stride

^b^Calculated using k2d3

### Characterization of hFliC filaments

**Fig. 2:**
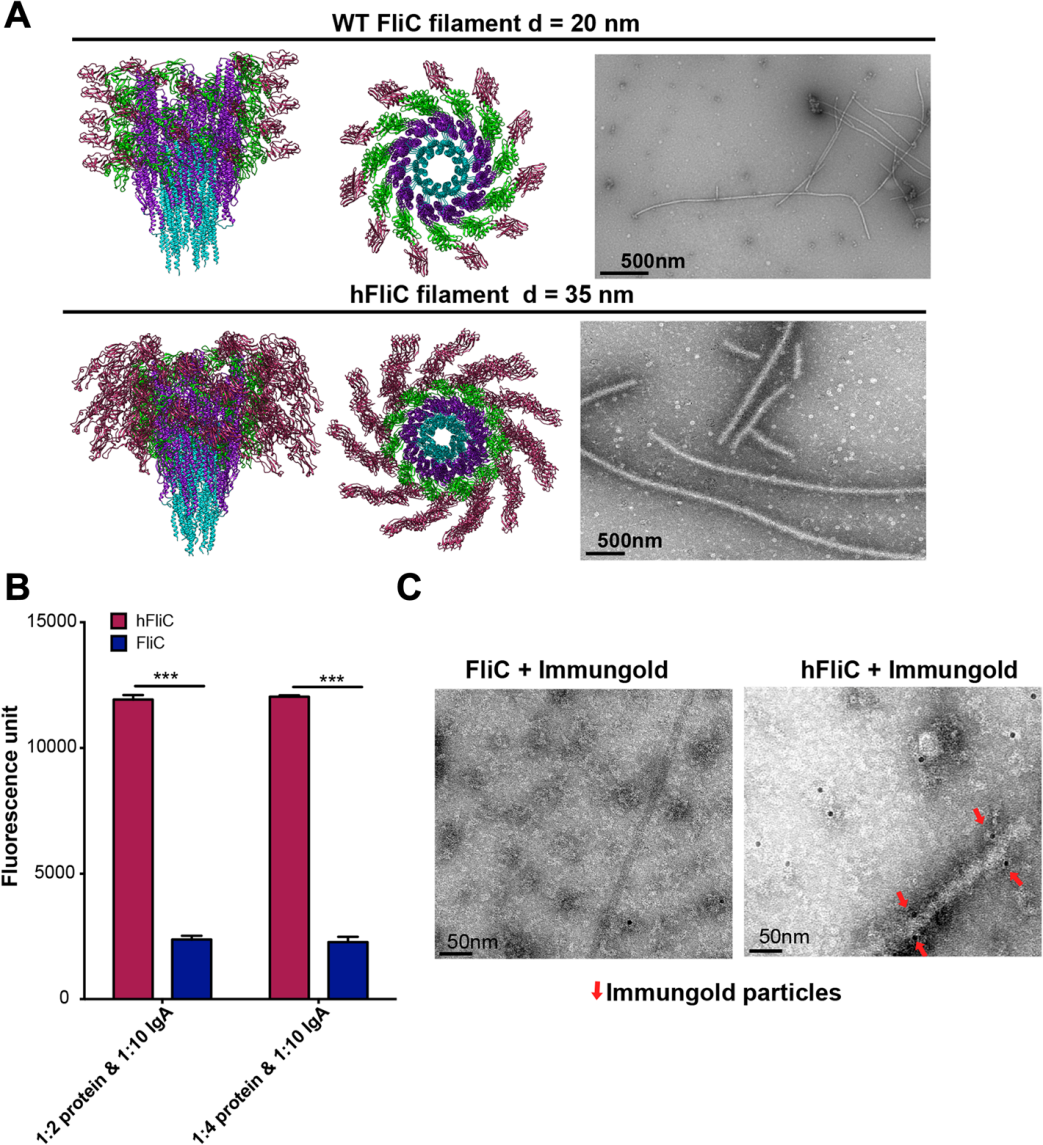
Characterization of hybrid flagellin filaments. **a** The top panel represents molecular structure of the parent FliC filament13 (left panel: XY view and middle panel: Z view). Negative stain TE micrograph of WT FliC filament generated by recombinantly produced FliC (right panel); FliC filament diameter was calculated to be 20 nm. The bottom panel represents a hypothetical molecular model of hFliC, where DENV2 + GS linkers were modeled into native FliC filament to replace D3 domain. The diameter of hFliC was computationally determined to be 35 nm. Negative stain TE micrographs confirmed resulting hFliC filaments had increased diameter compared to FliC filament. **b** Binding ELISA was performed using DENV2 specific monoclonal IgA antibody. hFliC showed binding to monoclonal IgA, compared to FliC alone, which showed no binding. Experiments were performed in triplicate (*n* = 3), and error bars represent mean +/- SEM, Student’s t-test was used to determine statistics, and a *p* < 0.05 were considered statistically significant. **c** Immunogold particle labeling against DENV2 E specific IgG (primary antibody, isolated from 4G2 culture supernatant), was used to determine the placement of the DENV2 E portion relative to the rest of the filament. Negative stain transmission electron micrograph of immunogold labeling of WT FliC filaments (left panel), confirming that no immunogold particles bound to WT filaments. Right panel is a negative stain transmission electron micrograph of immunogold labeling of hFliC filaments, which indicated that the immunogold particles primarily associate with the outside of the hFliC filament

A hypothetical molecular model of the hFliC filament predicts an independent domain of DENV E at the surface of an intact 11-protofilament polymer (Fig. 2a). Accordingly, we determined that the hFliC protein was able to form filaments in high molar ammonium sulfate or sodium citrate (Fig. 2a and data not shown), similar to the reported molar conditions for *in vitro* WT FliC polymerization [15]. To evaluate these filaments, we used negative stain transmission electron microscopy (TEM). Morphometric analysis of the filaments revealed an increased diameter of 35 nm compared to WT FliC filaments (diameter of ~20 nm [14]), consistent with the hypothetical molecular model of the hFliC filament (Fig. 2a). However, we do not have sufficient resolution to confirm that the hFliC forms the prototypical 11-protofilament core structure as suggested by the hypothetical molecular model.

The inner core of the hFliC filaments exhibited a high degree of contrast, comparable to Tobacco Mosaic Virus (TMV) filaments (known to have high contrast) (Additional file 1: Figure S2a,b). However, the outer regions of the filaments displayed a low degree of contrast relative to the rest of the filament suggesting that individual DENV2 antigen domains were able to rotate freely at the surface of the filament (further confirmed via cryoelectron microscopy, data not shown). This flexibility might be beneficial to the avidity of the DENV2 antigenic domain of the polymeric hFliC vaccine.

To confirm that the DENV2 E domain retained its antigenicity, we used a DENV2 specific recombinant IgA monoclonal antibody (Additional file 1: Figure S3a,b); this antibody was produced from sequences derived from the DENV2-specific 4G2 hybridoma because we discovered that the original hybridoma produced mixed antibody complexes, not all being specific for DENV2 E protein. We tested the recombinant IgA binding to control recombinant DENV2 E domain, as well as in the hybrid hFliC protein and polymerized hFliC filament. In an ELISA assay, the DENV2 E hFliC was able to bind the 4G2 IgA (Fig. 2b, negative control FliC alone).

More importantly, we also confirmed that DENV2 E epitopes were available on the surface of the polymerized filament. This was confirmed by immunogold labeling of the hFliC filament using DENV2 specific IgG produced by the 4G2 hybridoma (Fig. 2c). The 4G2 antibody is known to bind/recognize an epitope region in the extremity of domain DII (important for membrane fusion) of the DENV2 E protein; based on our hFliC molecular model, this is the domain most solvent exposed in filament formation (Fig. 1a: [22, 23]). Together, these data demonstrate 1) that the hFliC protein is able to form filaments in a similar manner to WT FliC, 2) the DENV2 E subunit inserted into the middle of FliC retains its conformational independence and 3) its antigenicity is retained and recognized by anti-DENV2 E-specific antibody.

### *In vivo* activity of hFliC in TD model

**Fig. 3:**
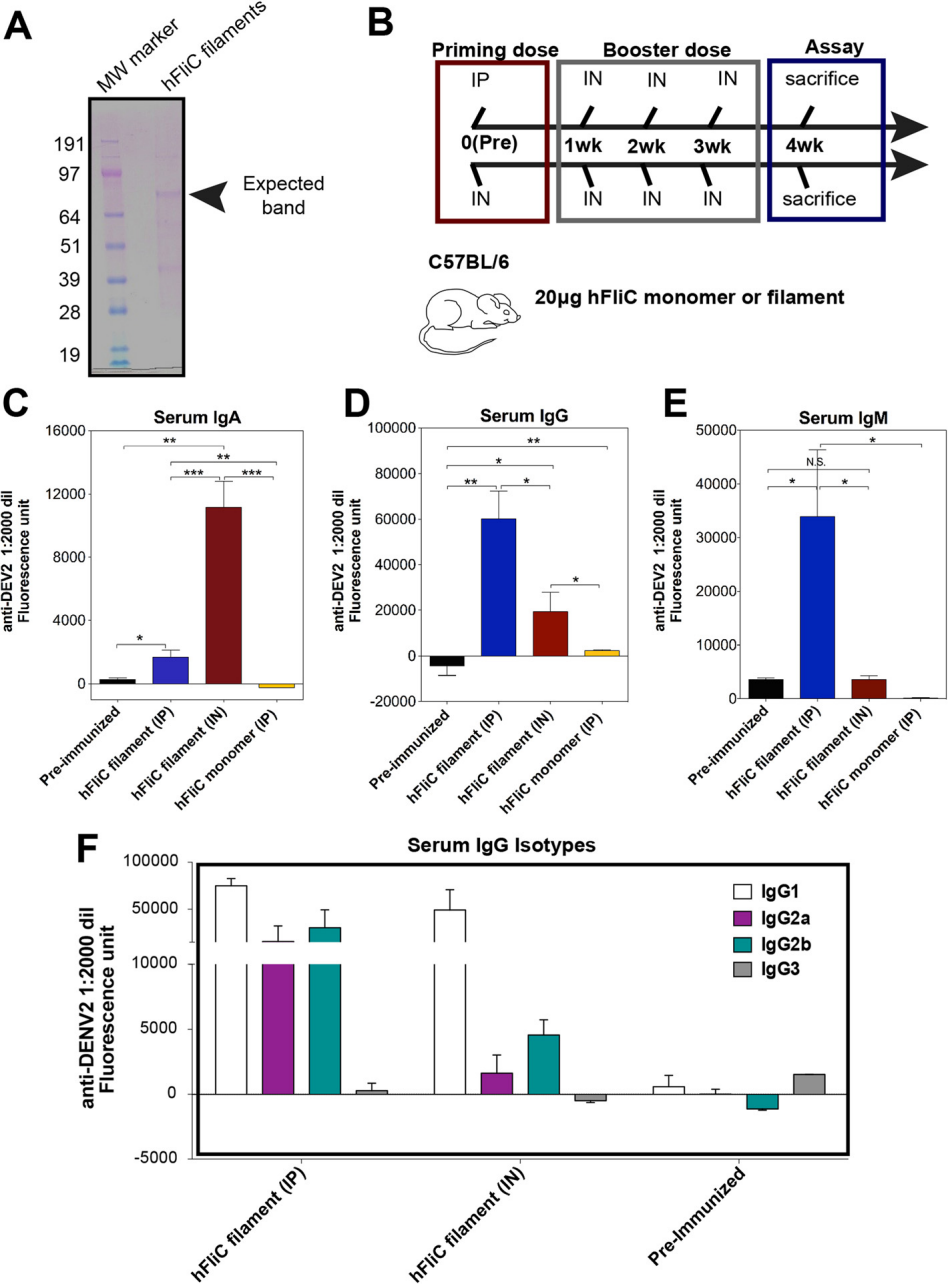
Humoral immune response in non-restricted mouse models. **a** Purified hFliC protein was polymerized in high molar ammonium sulfate and resulting filaments were pelleted with high-speed centrifugation and resuspended in 150 mM PBS. Under denaturing conditions the major band was only that of the full-length hFliC (**b**) Schematic diagram of *in vivo* immunization protocol used. The immunization period was five weeks, with four injections spaced a week apart. Priming injection at week zero was either administered IP or IN, and the three subsequent booster doses where all administered IN. **c** ELISA scans show serum IgA response in WT C57BL/6 J mice, assayed after the five-week immunization period. C57BL/6 J mice given polymeric hFliC IN throughout the entire immunization exhibited a strong serum IgA response, while all other mice showed no IgA titer. **d** ELISA scans for serum IgG response in WT C57BL/6 J, assayed after the five-week immunization period. C57BL/6 J mice given polymeric hFliC IP showed a stronger IgG response compared to mice given monomeric hFliC IP. **e** ELISA scans for serum IgM response in WT C57BL/6 J mice assayed after the five-week immunization period. Again, C57BL/6 J mice receiving polymeric hFliC IP showed strongest IgM response. **f** Serum IgG isotypes were assayed in C57BL/6 J mice, mice given polymeric hFliC IP showed a robust IgG1 response. All groups in immunization protocols used an *n* = 3-5 mice per group, and error bars represent the mean +/- SEM, and for statistical analyses, * - *p* < 0.05; ** - *p* < 0.005; *** - *p* < 0.0005. Values were reported as the fluorescence measured in 1:2000 serum dilutions, with background fluorescence values subtracted

Since we demonstrated that hFliC exhibited proper conformation and antigenicity in its polymerized form, we were ready to test its ability to stimulate an immune response *in vivo*. Using an immunization protocol similar to that used by Lo *et al*. [24] but in this case without the addition of any additional adjuvant (such as cholera toxin), we tested the immunogenicity of monomeric and polymeric hFliC in wild type mice (C57BL/6 J; Fig. 3b). We first administered a priming dose of hFliC filaments (Fig. 3a) or hFliC monomers, given either intraperitoneally (IP) or intranasally (IN), followed by three IN booster doses at one-week intervals for 3 additional weeks.

We found that hFliC filaments induced a stronger antibody response than their monomeric counterparts in C57BL/6 J WT mice (Fig. 3c-e and Additional file 1: Figure S4). IP administration of polymeric hFliC during the priming injection resulted in the highest serum IgG (total) and IgM responses (Fig. 3d,e and Additional file 1: Figure S4A,C). Mice given IN priming and booster injections of polymeric hFliC throughout the immunization protocol showed the strongest serum IgA response (Fig. 3c and Additional file 1: Figure S4B). Since different delivery routes gave different Ig responses in different tissues, we also tested whether these immunization protocols resulted in the production of different IgG subclasses (Fig. 3f). Thus, polymeric hFliC with either IP or IN priming induced strong serum IgG1 response, while the IP priming dose induced the best IgG2a and IgG2b response. In sum, the IN priming protocol induced both the best IgA and weakest IgG2a/b response, consistent with a Th2-type response. By contrast, the IP priming protocol induced the strongest IgG2 and IgM responses, though this would not be as easily categorized as a strict Th1-type response.

### hFliC filaments can crosslink BCRs

**Fig. 4:**
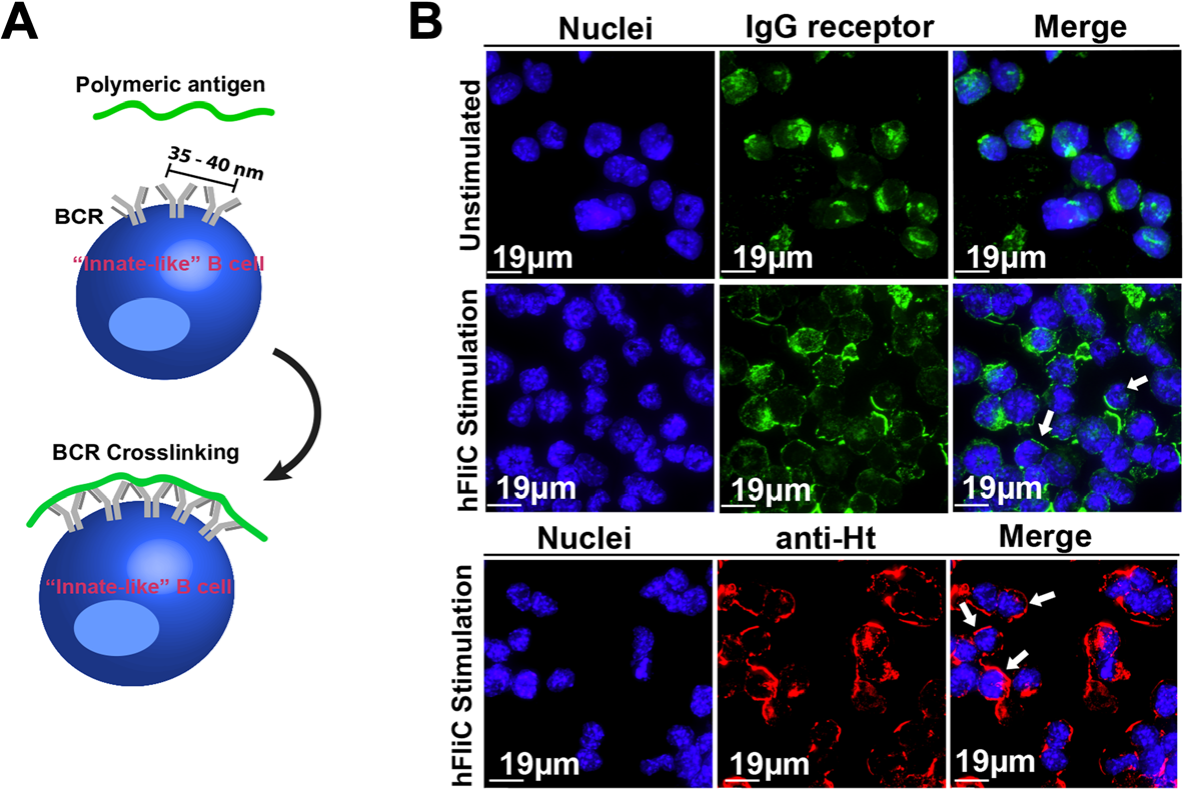
Hybrid flagellin filaments are able to crosslink BCR *in vitro.* **a** Schematic of how TI immune response is generated. Polymeric antigens bind to B cell receptors and crosslink them. Once this crosslinking event occurs, the B cell will self-differentiate into plasma cells to produce antibodies. **b** 4G2 B cell hybridoma (DENV2-specific IgG producing hybridoma) stimulated with polymeric hFliC. Stimulated cells showed more organized expression of Ig receptors compared to unstimulated cells, indicating cells were crosslinked (top panel). This was further confirmed by staining with anti-Histag antibody

TI responses rely on the ability of antigens to crosslink BCRs above a threshold number; this clustering promotes the T helper cell-independent differentiation of B cells into plasma cells (Fig. 4a). To determine whether the repetitive DENV2 E determinants displayed on hFliC filaments were capable of BCR crosslinking, we tested them on cells expressing receptors specific for the DENV2 E protein. The 4G2 B cell hybridoma expresses surface Ig receptors specific for DENV2 E protein, so these cells were incubated with hFliC filaments. Confocal micrographs of cytospin slides of unstimulated cells exhibited a randomized distribution of Ig receptors, while cells incubated with hFliC filaments exhibited a more organized distribution of the BCRs around the circumference of the cell (Fig. 4b, showing staining of surface Ig). This was further confirmed by staining with an anti-HisTag antibody (Fig. 4b), suggesting that the hFliC filament was in direct contact with the BCR receptors and these receptors had in fact been clustered by the hFliC filaments.

### *In vivo* activity of hFliC polymers in TCR restricted model

**Fig. 5:**
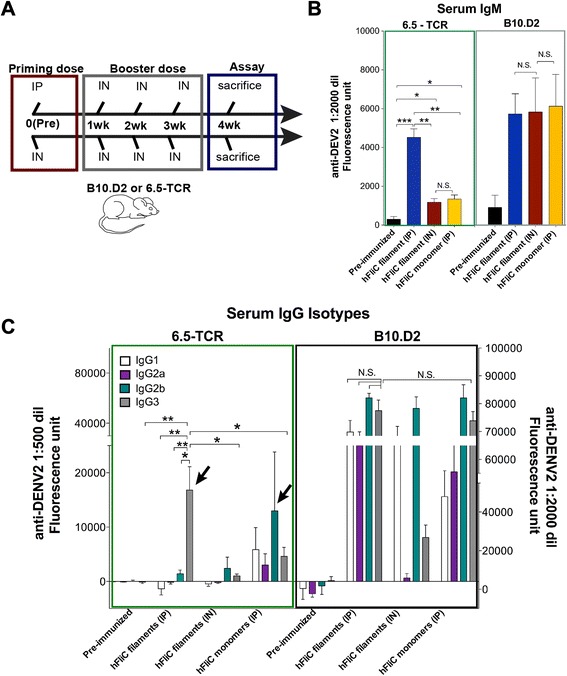
Humoral immune response in TI mouse model (6.5-TCR transgenic). **a** Schematic diagram of *in vivo* immunization protocol used. The immunization period was five weeks, with four injections spaced a week apart. Priming injection at week zero was either administered IP or IN, and the three subsequent booster doses were administered (IN). **b** ELISA scans of serum IgM in 6.5-TCR and B10.D2 over five-week immunization period. Only mice given polymeric hFliC IP in the priming dose showed a serum IgM response. All groups B10.D2 mice immunized showed a strong IgM response. Statistics were determined by unpaired Student’s t-test, *p* < 0.05 were considered statistically significant, and error bars represent the mean +/- SEM. **c** ELISA scans of serum IgG isotypes in 6.5-TCR, and B10.D2 mice. 6.5-TCR mice given polymeric hFliC IP in the priming dose exhibited a specific IgG3 response, meanwhile B10.D2 mice showed broader subclass distribution (more balanced between Th1 and Th2). All groups in immunization protocols used an *n* = 3-5 mice per group, and error bars represent the mean +/- SEM. Values were reported as the fluorescence measured in 1:2000 serum dilution in the case of IgM and IgG isotypes for B10.D2 immunized mice, and values were reported as the fluorescence measured in 1:500 serum dilution for IgG isotype for 6.5-TCR immunized mice, with background fluorescence values subtracted, and for statistical analyses, * - *p* < 0.05; ** - *p* < 0.005; *** - *p* < 0.0005. Arrows show that there was a specifically increased IgG3 response in 6.5-TCR mice to IP hFliC filament while IgG2b responses were higher to monomer, while by contrast, in B10.D2 mice IgG1 and IgG2a responses were strong to both IP hFliC filament and monomer

To test the potential for the polymeric vaccine to induce TI responses, we performed *in vivo* immunization in a T cell repertoire-restricted model (Fig. 5a). Here we used a T cell receptor transgenic mouse model (6.5-TCR, recognizing an influenza hemagglutinin peptide presented on MHCII I-E^d^: [25, 26]), in which nearly all CD4 T cells express the mono-specific hemagglutinin-specific antigen receptor. Allelic exclusion during thymic selection would prevent the development of T cells expressing any additional antigen receptors. This particular transgenic mouse model was chosen because the degree of allelic exclusion is more potent in this model compared to most other models, obviating the need for including additional targeted genes (e.g., Rag1) that would cripple the immune system. As a result, the monospecific CD4 T cell population only recognizes an antigen from influenza not seen under normal conditions, and so any CD4 T helper cells would be unable to provide cognate antigen-specific help to FliC- or DENV-specific B cells. Since a relatively normal number of T cells are still produced, all other components of the immune system, including organized lymphoid tissues, would still develop normally. Since the 6.5-TCR mice were on a B10.D2 background, B10.D2 mice were used as WT controls.

With an immunization protocol using hFliC filaments administered IP (priming dose), 6.5-TCR mice were still capable of producing a strong serum IgM response within the range of B10.D2 controls (within a two-fold dilution); no detectable levels of IgA were observed in any condition (Fig. 5b and Additional file 1: Figures S5a-c). Interestingly, B10.D2 mice treated with polymeric hFliC in three IP doses exhibited the most robust IgM and IgG of any group of mice used in this study (Additional file 1: Figure S5D), raising the possibility that this protocol was particularly effective in targeting peritoneal B1 cells. Finally, we wanted to determine whether differences existed in the IgG isotypes produced in wild type B10.D2 or the TCR restricted (6.5-TCR) mice, as the IgG3 subclass has been previously identified as more prominent in TI antibody responses [9]. B10.D2 mice showed a robust Th1 serum IgG2b response, with a lower serum IgG3 response (Fig. 5c and Additional file 1: Figure S5D). Interestingly, although IgG titers were lower in the 6.5-TCR mice compared to B10.D2 mice due to the absence of cognate T cell help, 6.5-TCR mice given hFliC filament IP showed a significant IgG3 response (Fig. 5c).

## Discussion

Taken together, these data suggest that hFliC filaments are indeed capable of inducing a significant antibody response in a T cell restricted model. Development of vaccines with multivalent binding (clustering) has largely been accomplished by the linkage of two proteins in a linear fashion; however, this method has had limited success in certain diseases [27–29]. Thus, the reengineering of FliC by replacing its solvent exposed FliC D3 domain with a conformationally intact viral envelope protein domain may hold promise for an entirely new vaccine framework. This may be especially important in the development of vaccines for patients with less robust immune systems, including those in the early perinatal period, in people suffering from various T cell immunodeficiency disorders (HIV-AIDS, DiGeorge syndrome), patients undergoing chemotherapy, as well as elderly patients with sluggish adaptive immunity.

The increased potency of the hFliC relative to the monomeric form may relate to multiple factors. In the polymeric form the antigen may be more able to cross-link the antigen-specific B cell receptors. As a less soluble complex the filament may also be more easily taken up by antigen presenting cells for stimulation of T helper cells. In addition, the polymeric antigen may also have a greater potential to recruit the T helper-independent B-1 B cell population; this is evidenced by the strong IgM and IgG3 response in the 6.5-TCR mice when hFliC filaments were injected into the peritoneum, where B-1 B cells are dominant. More detailed studies on the IgM (and IgG3) response will be helpful in determining any benefit in recruiting B-1 B cells in protective immunity, as it may help recruit a distinctive antibody repertoire.

To date no licensed vaccine exists for DENV infection. This is complicated by the concern that sub-neutralizing levels of cross-reactive anti-DENV antibodies might result in more severe disease (i.e., Dengue Hemorrhagic Fever; [30–34]. Thus, it is crucial to develop a vaccine framework that is equally effective against all DENV serotypes. Live attenuated tetravalent viruses have shown only limited success in both non-human primates and humans, but it has been difficult to establish a proper balance between immunogenicity and adverse reactions with these formulations [35, 36]. In addition, while recombinant DENV subunit vaccines can induce neutralizing titers in mice when co-administered with adjuvant [37], these were found to be less efficacious. Our approach of reengineering FliC to replace its solvent exposed FliC D3 domain with antigenic determinants allows us to combine the benefits of the immunological adjuvant effects of flagellin with the added immunogenicity of polymeric vaccine antigens. Moreover, independent replacement of the FliC D3 domain with envelope proteins from each of the four DENV serotypes, and then producing mixed filaments with stoichiometric amounts of each hFliC protein could produce a universal DENV vaccine with balanced broadly neutralizing capabilities (i.e. against all serotypes). This strategy may overcome the problem noted in a recent trial with a tetravalent recombinant vaccine, in which the response to one serotype was much lower than the response to the other serotypes [38].

## Conclusions

We reengineered the native FliC structure by replacing the FliC D3 domain to contain a conformationally intact DENV2 E protein domain, without compromising either protein’s structure. Moreover, this hybrid protein could be polymerized to produce filaments capable of stimulating a humoral antibody response; filaments were found to be more potent induces of antibody responses than monomers. More importantly, these hybrid protein filaments were capable of crosslinking B cell antigen receptors *in vitro* and accordingly were also shown to induce significant antibody responses even in a T cell repertoire-restricted transgenic mouse model. In sum, this study lays the groundwork for the use of FliC as a vaccine scaffold for development of novel vaccines for use even in immunodeficiency settings.

## Methods

### Design of hFliC protein

Flagellin was PCR amplified from an earlier vaccine design, where the DENV2 portion was linked to FliC at the N’ terminal domain through a GS linker. This FliC was first PCR amplified from genomic DNA isolated from a culture of *S. Typhimurium*. Flagellin was PCR amplified in two fragments. The first piece was amplified using a forward primer with flanking 5′ BamH1 site and reverse primer with 3′ flanking Kpn1, EcoR1 site, that had amplified only the first 184 residues. The second piece was amplified using a forward primer with 3′ flanking Kpn1, EcoRI site and reverse primer with 5′ Xho1 site, which amplified residues 285–494 plus a 6x-His tag. These two pieces were then PCR overlapped. DENV2 E sequence from a mouse adapted strain [39] was PCR amplified from the initial clone, and flanking N’ and C’ terminal GGGSGGGS linkers were then ligated into FliC lacking D3 via the Kpn1 and Ecor1 sites. Gene constructs were checked for appropriate sequence throughout the cloning process. This final construct was then ligated into the pENTR 3C (life technologies) expression vector for recombination and subsequent expression.

### Production and purification of recombinant proteins (hFliC, FliC and DENV2 E)

Recombinant protein was produced using Baculovirus expression system. First, cloned cDNA construct was transfected into SF21 insect cells (Life Technologies) in Grace’s supplemented or unsupplemented media (Thermo Scientific) based on manufacturer’s protocol. Resulting protein viral stocks were then transduced into SF9 insect cells (Life Technologies), cultured in HyClone SFX-Insect media (Thermo Scientific), for larger scale production. The insect cells secreting the protein were cultured at 27 °C, and the supernatant was harvested after an optimized incubation period. The native FliC contains a signaling domain that allows the protein to be secreted, and this signaling domain was retained in the hFliC protein. As DENV2 E is a transmembrane protein, a leader peptide was added to it so that it would be secreted into the culture supernatant. The insect culture media was filtered before purification. Western blot (Life Technologies) was used to verify protein expression and purity before precipitating protein from the media with 80 % saturated ammonium sulfate (Fisher). After a 2 h precipitation at 4 °C, protein was centrifuged at 12,000 rpm for 30 mins to pellet the protein. The protein pellet was resuspended with 1XPBS + 0.05 % Tween-20 and dialyzed over night in a porous membrane with a molecular weight cut off of 12–14,000 (SpectrumLabs). Protein was then bound to HisPur cobalt resin (Thermo Scientific) overnight. Bound protein was eluted from HisPur resin using increased concentrations of imidazole. Eluted protein was subjected to a final dialysis to rid the protein sample of any traces of Tween-20 and imidazole. The final protein was concentrated using polyethylene oxide (PEO: Sigma Aldrich). Concentrated protein was checked for purity using both Coomassie (Thermo Scientific) and Western blot (1 ° antibody rabbit-α-Ht (Santa Cruz Scientific) and 2 ° Donkey α-rabbit @680 nm (Odyssey)), and concentration was quantified either using BCA or Bradford.

### Molecular modeling of FliC and hFliC filaments

The structure of FliC (PDB code 3A5X) and DENV2 (PDB code 1OAN) were obtained from the Protein Data Bank. The hFliC filament was built using the molecular structures of the FliC filament and the DENV2 protein. The D3 domain of a FliC monomer was deleted (residues 185–285), and the coordinates of DENV2 were manually translated and rotated until the N- and C-termini of DENV2 were in close proximity to the C- and N-termini of the end of the D2 domain of FliC. The linkers between FliC and DENV2 (sequence GGGSGGGS) were added using Modeller [40].

### Polymerization of hFliC or FliC

Based on previous studies, purified FliC or hFliC proteins samples were diluted in either 3.0 M ammonium sulfate or sodium citrate [15, 41]. A final buffer concentration of 1.5 M was obtained for each buffer + protein sample. This molar condition has been suggested to be ideal for FliC polymerization. Each buffer + protein sample was allowed to polymerize overnight at room temperature. Polymers were pelleted with high-speed centrifugation, and washed in with 150 mM PBS. These samples were then used for either TEM or vaccination.

### Circular dichroism

Circular dichroism was used to determine secondary structure of the hFliC protein. Spectra were collected for each protein (FliC, DENV2, hFliC) using Jasco J-815 CD Spectrophotometer. Measurements were performed at 25 °C in a 1 cm path length cuvette (Varian), over a wavelength range of 190–260 nm. Protein for circular dichroism measurements was diluted in water to eliminate the effects of NaCl absorption, which absorbs strongly at wavelengths below 190 nm.

### Negative stain transmission electron microscopy

For transmission electron microscopy studies, formvar carbon coated grids (Ted Pella) were glow discharged for at least 20 s, 3 μl of polymeric sample was then loaded onto formvar carbon coated grid, and excess liquid was removed. Grids were then washed 3 times in PBS. For viewing in TEM, grids were negatively stained with either 4 % uranyl acetate or 2 % PTA (phosphotungsten acid) and placed into desiccator until imaged. Micrographs were obtained using JEM-1011 transmission electron microscope or PHILIPS TECNAI 12 transmission electron microscope.

### ELISA of hybrid flagellin

ELISAs were performed to see if the hFliC was capable of binding DENV2 specific antibodies. Briefly, ELISA plates were coated with either purified hFliC or purified FliC (negative control) at two different dilutions (1:2 and 1:4) and incubated overnight at 4 °C. Plates were then washed, blocked and DENV2 specific monoclonal IgA antibody (at two dilutions) was added and again incubated overnight. Detection was performed by adding anti-IgA antibody (Southern Biotech) conjugated to Alkaline Phosphatase, and 4-MUP (Life Technologies) was added for detection by fluorescence.

### Design of DENV2 recombinant IgA

We discovered that the 4G2 hybridoma produced 4 different Ig transcripts and multiple peptides, but only one pair specific for DENV2 E binding. Mass spectrometry N-terminal protein sequencing results of purified D1-4G2-4-15 hybridoma (4G2; ATCC# HB-112) antibody was used to determine the leader peptide nucleotide sequence of the correct variable regions. These 4G2 Dengue-specific kappa light and IgG2a heavy chain sequences were cloned and deposited in Genbank; NCBI accession code for kappa light chain is KJ438784 and IgG2a heavy chain is KJ438785. The full-length kappa sequence was amplified from 4G2 cDNA and cloned into pCDNA3.1(+) (Life Technologies) using NheI and EcoRI sites. The heavy chain variable region amplified from 4G2 hybridoma cDNA was overlapped with the IgA heavy constant region amplified from BALB/c spleen cell cDNA [22]. GS linker and 6XHis-tag PCR fragment was added with the using another round of overlap PCR. The complete Dengue specific IgA heavy chain was cloned into pCAG-eGFP-puro by replacing the eGFP with Age1 and Not1 before ligation and transformation [42]. The Dengue specific IgA heavy chain clone was confirmed by sequencing.

### Production and purification of DENV2 recombinant IgA

Dengue specific kappa light and IgA heavy chain DNA constructs was stably transfected into the Chinese hamster ovary cell line (CHO; CHO-K1; ATCC# CCL-61) and clonally selected via limit dilution under selection media (F-12 K media (Corning) supplemented with 10 % fetal bovine serum (Biowest), 1000 μg/ml G418 (Corning), and 10 μg/ml puromycin (Sigma)). Final highest IgA-producing clone was adapted to serum free media (Corning) and used for production. The final clone was expanded in 40-T150-flasks and overgrown for one week prior to supernatant collection in serum free media. One liter of the supernatant media total protein was used to purify the rIgA (purification step as mentioned above). The production yield and purity was determined by Western blot, Coomassie, DENV2 protein binding ELISA, and total IgA ELISA quantification.

### Immunogold labeling of hybrid flagellin filaments

Immunogold labeling was performed to further confirm that the DENV2 subunit was presented on the surface of the filaments. Here, filaments were loaded onto carbon coated formvar grids. The grids were then incubated in 1XTBST (tris-buffered saline with Tween) with 0.05 % BSA (Bovine serum albumin) for 30 min. Grids were washed and incubated with 4G2 (Dengue specific IgG hybridoma) for two hrs; again the grids were washed and incubated with Goat anti-mouse IgG conjugated gold particles (10 nm: Ted Pella). Finally, the grids were stained with 2 % uranyl acetate and viewed.

### B cell crosslinking

Crosslinking studies were performed using a 4G2 B cell hybridoma (ATCC# HB-112). 4G2 cell line was cultured/maintained, based on ATCC recommendation, using Dulbecco’s modified Eagle’s medium (4 mM L-glutamine, 4.5 g/L of glucose,1.5 g/L sodium bicarbonate, and 10 % fetal bovine serum: Cellgro). Cells were maintained at a concentration 1 × 10^6^ cells/ ml, and were passed every three days. To assess if hFliC filaments were able to crosslink the BCRs of the 4G2 cell line, cells were first stimulated with a 20 μg dose of hFliC filaments (prepared as mentioned above) for 30 mins under ambient conditions. After the 30 mins incubation, cells were washed 3X in 1XPBS. Cells stimulated with hFliC filament antigen as unstimulated control were then spun onto glass slides (Superfrost plus, Fisher Scientific) using a Cytospin (Shandon). Cells were then fixed with 1 % Paraformaldehyde/PBS and then permeabilized first with cytoskeleton buffer, followed by 0.5 % Tween-20/PBS and washed with 3X 0.1 % Tween-20/PBS. Cells were then blocked with 0.1 % Tween-20 Casein solution (Thermo Scientific) for at least 30 mins. Cells were then stained with anti-Histag (Santa Cruz Biotechnology) polyclonal IgG antibody (@488 nm) for at least 1 h, and again were washed. For cells stained with anti-histag antibody, these cells were again washed and secondary antibody was added (Donkey anti-rabbit @ 647: Life technologies) Finally, cells were post fixed with 4 % PFA/PBS solutions and mounted with Prolong Gold antifade reagent (Life Technologies), which contained DAPI to counterstain the nuclei. Images were obtained using a spinning disk BD CARVII Confocal Imager (BD Biosystems) attached to a Zeiss Axio Observer inverted microscope. Hardware, including microscope, confocal and digital camera (Qimaging Rolera EMC^2^) was controlled using Metamorph imaging software. Images were further optimized using Volocity deconvolution software.

### Immunization protocol

C57BL/6J, B10.D2, 6.5-TCR (Tg(Tcra/Tcrb)1Vbo) mice were maintained under specific pathogen free colony conditions at the UC Riverside vivarium. All procedures were performed in accordance with UCR Institutional Animal Care and Use Committee (IACUC) and NIH guidelines. Immunizations were performed over a five-week period, where the first dose (priming dose) was administered either through intranasal (IN) or intraperitoneal (IP) injections. For intranasal injections, a volume of 20 μl containing 20 μg protein was administered (10 μl containing 10 μg of protein into each nostril). For intraperitoneal injections, a volume of 200 μl, again with a total protein concentration of 20 μg, was injected into the peritoneum of mice using a 25 gauge needle. It should be noted all protein samples were diluted in 1XPBS to its final protein concentration. All mice were first anesthetized prior to injection. Three subsequent booster doses were given to all groups of mice via IN injections. Serum titers were assayed from peripheral blood collected by at the time points indicated. Mice were humanely sacrificed under anesthesia and cervical dislocation at the conclusion of the experiment.

### ELISA for quantification of antibody titers

Black flat-bottom plates (Costar) were coated with 10 μg/ml recombinant DENV2 (prepared as described earlier), in coating buffer (25 mM Na_2_CO_3_, 75 mM NaHCO_3_, pH 9.5). Plates were then washed 3X using 1X TBST buffer (50 mM Tris pH 7.5, 0.28 M NaCl, 6 mM KCl, 0.1 % Tween 20), using a Biotek ELx405 Automated Plate Washer. Plates were blocked for 2 h at room temperature in 3 % normal goat serum (Vector Labs). Samples were first diluted in blocking solution; serum was diluted 1:2000 and feces were diluted 1:10. Diluted samples were then two-fold serially diluted, and added to coated/blocked plated in triplicate. After washing, detection was performed with either Rat anti-mouse IgA-AP (Southern Biotech, diluted 1:1000), Goat anti-mouse IgM-AP (Southern Biotech, diluted 1:1000), Goat anti-mouse IgG-AP (Southern Biotech, diluted 1:2000), Goat anti-mouse IgG1-AP (Southern Biotech, diluted 1:1000), Goat anti-mouse IgG2A-AP (Southern Biotech, diluted 1:1000), Goat anti-mouse IgG2B-AP (Southern Biotech, diluted 1:1000), Goat anti-mouse IgG3-AP (Southern Biotech, diluted 1:1000), in 1X TBST. For final development, 10 mM 4-MUP (Molecular Probes) in DMSO diluted 1:25 in substrate buffer (50 mM K_2_CO_3_ 2 mM MgCl_2_, pH 9.8) was added. Fluorescence was detected 60 min later 360 nm excitation and 460 nm emission wavelengths on Molecular Devices SpectraMax M2e plate reader. Raw fluorescence values from the dilution series were then subtracted from background fluorescence values.

### Statistical tests

In general, groups were tested using Student’s t-test, as indicated for each experiment in the figure legends, with a *p* < 0.05 considered to be a significant difference for reporting. In the case of ELISA assays, Mann-Whitney tests were also used with similar results. In figures, * - *p* < 0.05; ** - *p* < 0.005; *** - *p* < 0.0005. Prism (GraphPad) software version 5.04 was used for statistical test calculations.

### Human subjects

No research in this report involved human subjects, human material or human data.

## Additional file

Additional file 1: (PDF 10340 kb)

## Abbreviations

TI: T cell-independent
FliC: Flagellin
DENV2: Dengue virus 2
hFliC: hybrid FliC protein
TD: T cell-dependent
BCR: B cell receptor
Th: T helper
TLR5: Toll-like receptor 5
NLRC4: Nod-like receptor CARD domain-containing protein 4
E: Envelope glycoprotein
TEM: Transmission electron microscopy
TMV: Tobacco Mosaic Virus
IP: Intraperitoneally
IN: Intranasally
PEO: Polyethylene oxide
PTA: Phosphotungsten acid
