# Supplementary material for: Hybrid flagellin as a T cell independent vaccine scaffold
Source: BMC Biotechnol. 2015 Aug 12;15:71. doi: 10.1186/s12896-015-0194-0 (PMC4534063; doi:10.1186/s12896-015-0194-0)
Supplement: Additional file 1: — (PDF 10340 kb) [file 12896_2015_194_MOESM1_ESM.pdf]

Additional file 1

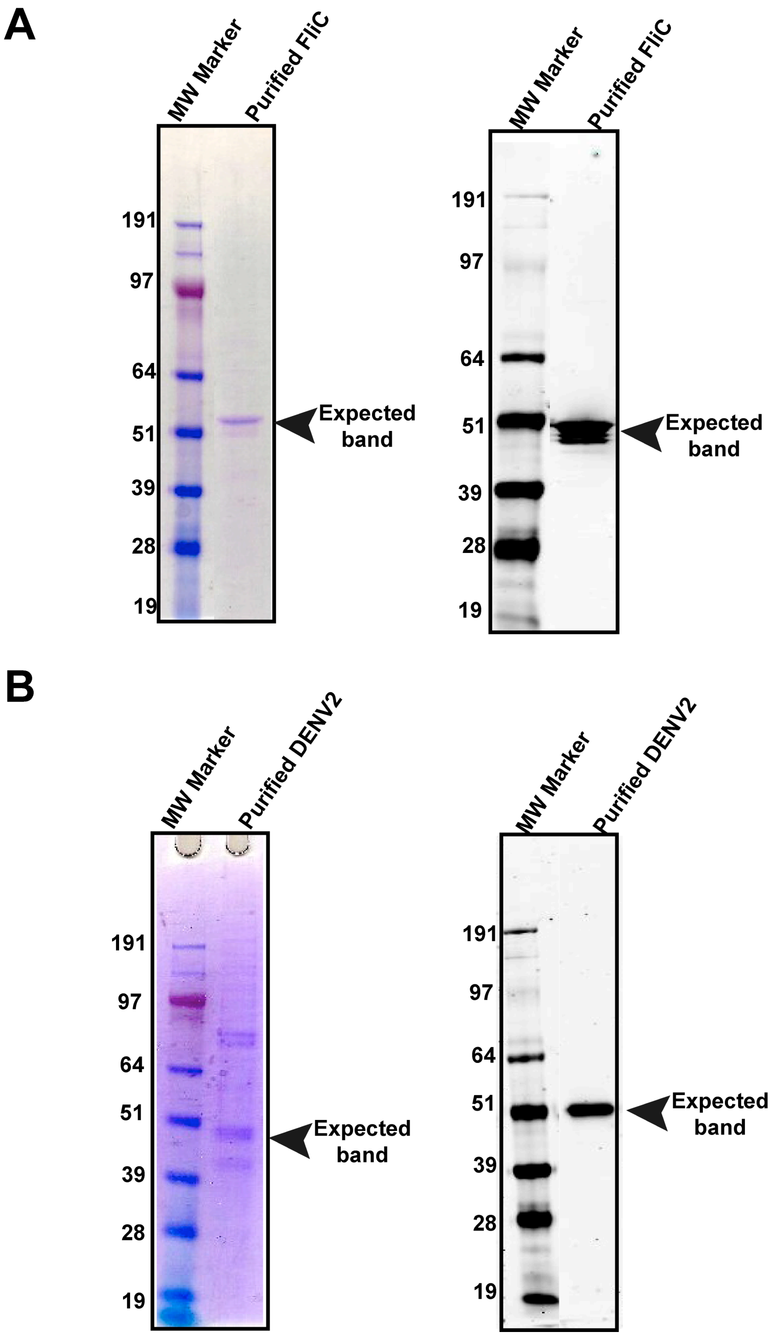

Figure S1. Production of purification of recombinant FliC and DENV2. (A) Coomassie (left panel) and Western blot against the C-terminal His tag were used to assess purity and size (56 kDa) of recombinantly produced FliC protein. Both Coomassie and Western blot confirmed FliC size as well as its relative purity, though some degradation was evident. (B) Analysis to confirm DENV2 purity and size (50 kDa) and revealed recombinantly produced DENV2 had appropriate size.

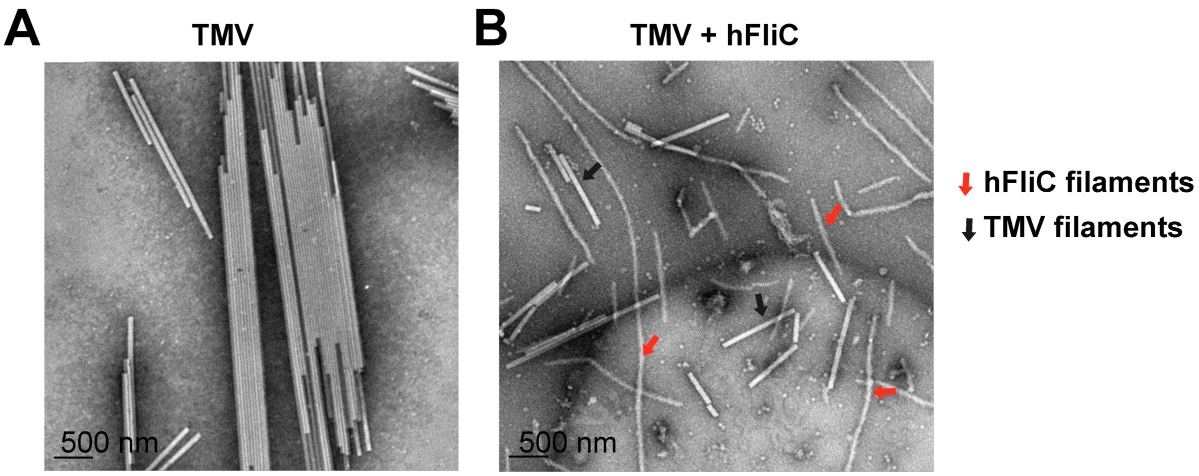

Figure S2. Further characterization of hFliC filaments. (A) Negative stain transmission electron micrograph of TMV (tobacco mosaic virus), which was used as a positive control for contrast (high degree of contrast). (B) Negative stain transmission electron micrograph of hFliC filaments compared directly to TMV filaments. The micrograph indicates the inner core the hFliC filaments have a high degree of contrast, while the outer portion (i.e. DENV2) lacks contrast.

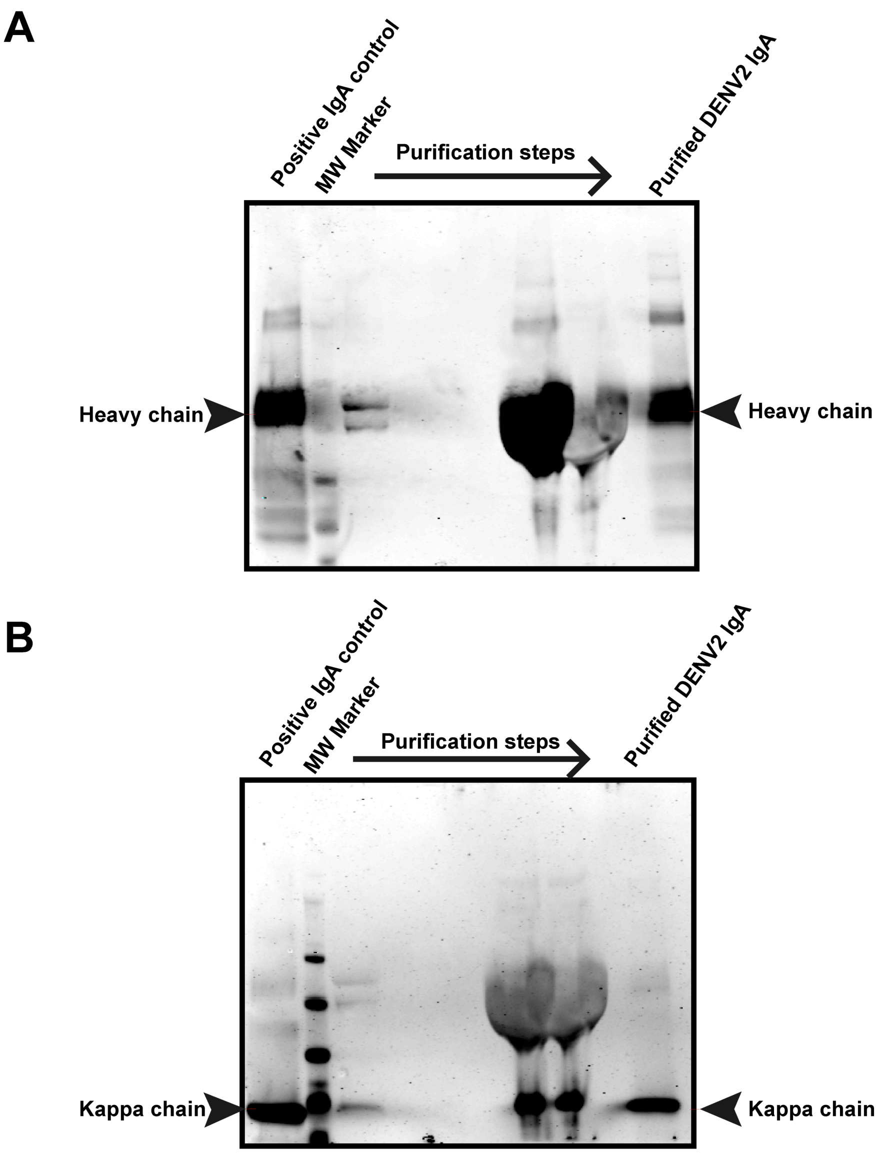

Figure S3. Recombinantly produced DENV2 specific monoclonal IgA**.** Positive IgA control used was purified mouse IgA, Kappa isotype control**.** (A) Western blot probing for IgA heavy chain blot indicates that DENV2 monoclonal IgA has anticipated size as compared to positive control. (B) Western blot probing for IgA kappa (light) chain, again blot indicates DENV2 monoclonal IgA has appropriate size as compared to positive control.

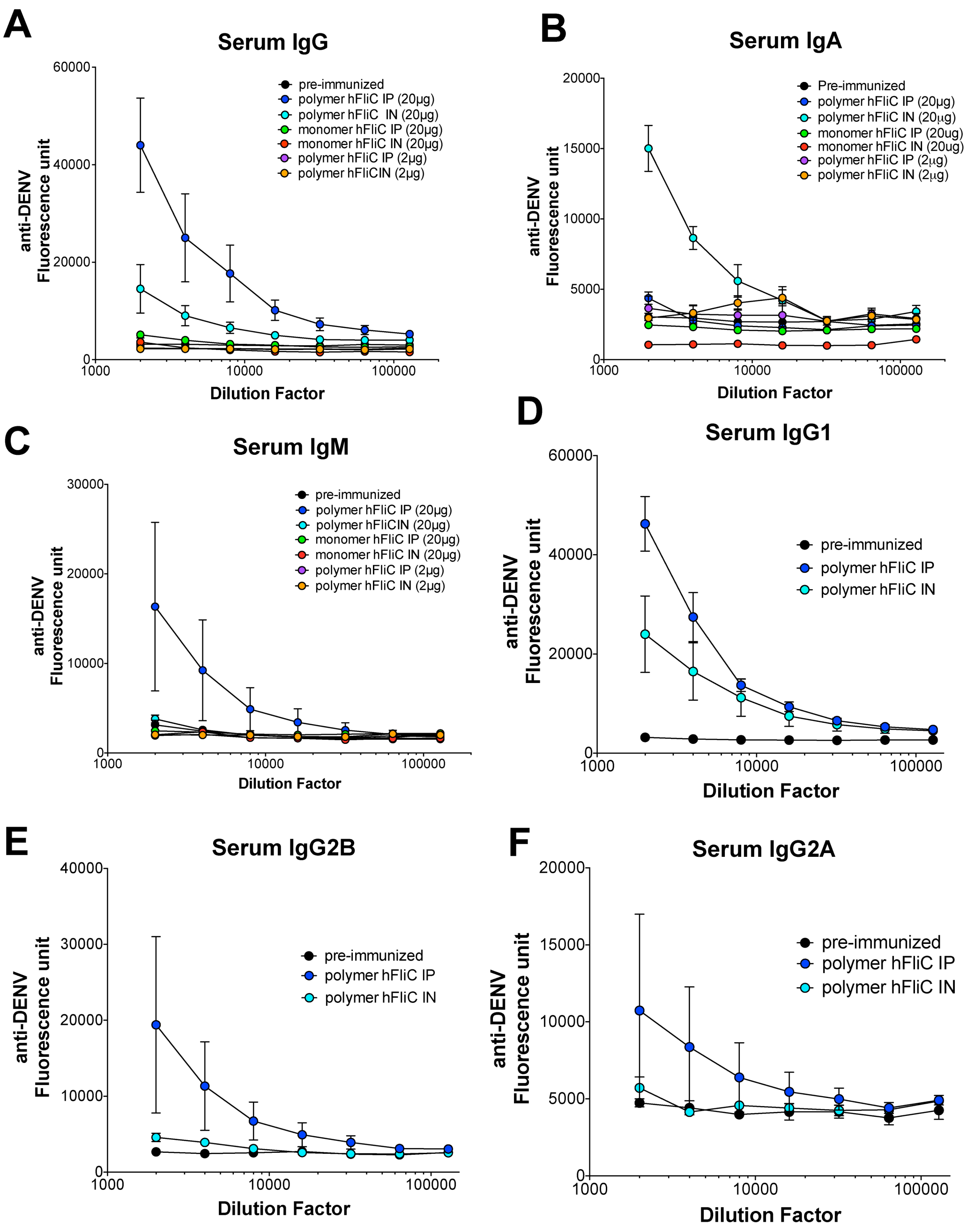

Figure S4. ELISA curves from immunized C57BL/6J mice, with either 20 or 2μg dose (which showed no response). All curves show the dilution series that was used. These values were subtracted from background fluorescence's and in most instances the 1:2000 dilution were used to make comparisons between groups. Same analysis was used for B10.D2 and 6.5-TCR immunized mice (but not shown).

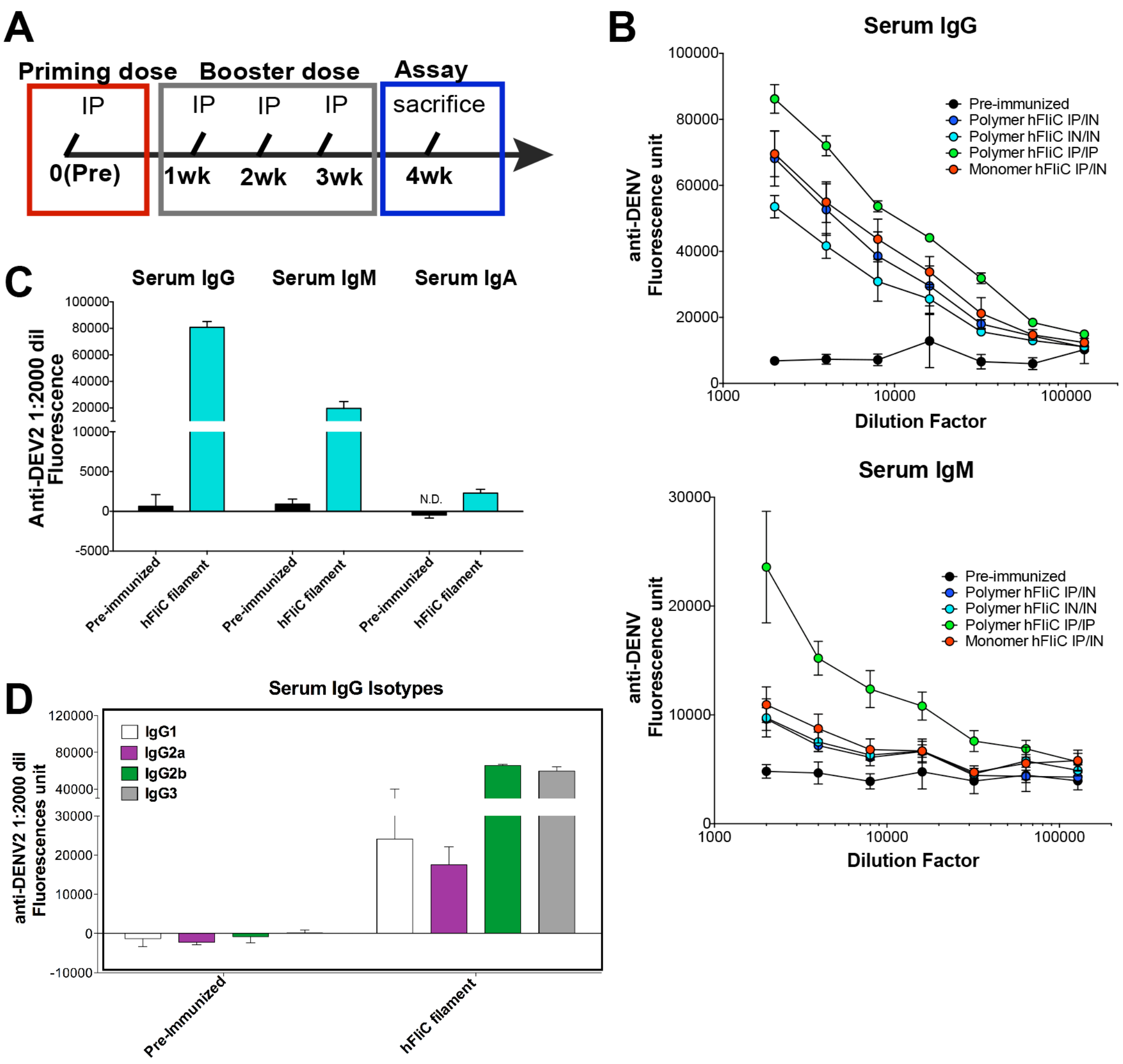

Figure S5. B10.D2 mice immunized with hFliC administered via IP injections. (A) Schematic of the immunization protocol that was used where IP injection was not only used in priming injection but also in all subsequent injections. B10.D2 mice given on hFliC filaments IP showed strongest IgG and IgM compared to all mice used in this study (B) Represents the raw dilution series, also comparing other immunization protocols, showing that the IP protocol induced the strongest IgM and IgG response. (C) Represents the response after background had been subtracted. (D) Mice exhibited a broad subclass IgG isotype response.
